# An exploratory study to characterize the HIV testing-to-care continuum to improve outcomes for Black and Latinx residents of South Los Angeles

**DOI:** 10.1371/journal.pone.0268374

**Published:** 2022-08-30

**Authors:** Breann M. McAndrew, Noemi Gil, David P. Lee, Senait Teklehaimanot, Katrina M. Schrode, Shanelle Bailey, Wilbert Jordan, LaShonda Y. Spencer, Ellen Rothman, Nina T. Harawa, Joseph Daniels

**Affiliations:** 1 Charles R. Drew University of Medicine and Science, Los Angeles, California, United States of America; 2 Martin Luther King Jr. Outpatient Center, Los Angeles, California, United States of America; 3 David Geffen School of Medicine, UCLA, Los Angeles, California, United States of America; 4 Edson College of Nursing and Health Innovation, Arizona State University, Phoenix, Arizona, United States of America; University of Arkansas for Medical Sciences, UNITED STATES

## Abstract

**Background:**

South Los Angeles (SPA6), with mostly Black (27.4%) and Latinx (68.2%) residents, has the second highest rates of new HIV diagnoses (31 per 100,000) in Los Angeles County. However, there is limited understanding of the HIV testing-to-care continuum among newly diagnosed in this setting.

**Methods:**

We conducted an exploratory study that analyzed de-identified data, including demographic characteristics and biomedical outcomes, from the electronic medical records of individuals newly diagnosed with HIV from 2016–2020 at the only public safety-net, county-run health department HIV clinic in SPA 6. We used Pearson Chi-square and Fisher’s Exact test to explore associations with HIV outcomes and a Kaplan-Meier survival curve to assess the time to linkage to care.

**Results:**

A total of 281 patients were identified. The majority (74.1%) presented with a baseline CD4 <500, many of which presented with a CD4<200 (39.2%). We found twice as many newly diagnosed Black individuals in our study population (48.2%) when compared to LAC (23%), despite only accounting for 27.4% of residents in SPA 6. The majority were linked to care within 30 days of positive test and prescribed anti-retroviral therapy. Viral suppression (59.8%) and undetectable VL (52.6%) were achieved within the year following diagnosis, with 9.3% lost to follow-up. Of those who became virally suppressed, 20.7% experienced viral rebound within the year following diagnosis.

**Conclusion:**

The large proportion of patients with a baseline CD4 <500 raises concerns about late diagnoses. Despite high rates of linkage to care and ART prescription, achievement of sustained viral suppression remains low with high rates of viral rebound. Longitudinal studies are needed to understand the barriers to early testing, retention in care, and treatment adherence to develop strategies and interventions with community organizations that respond to the unique needs of people living with HIV in South Los Angeles.

## Introduction

Despite significant advances in HIV prevention and treatment, disparities persist along the HIV care continuum for Black and Latinx people [1–7]. Between 2010 and 2016, new HIV diagnoses increased by 30% among Latinx gay, bisexual, and other men who have sex with men [8]. HIV incidence for men who have sex with men (MSM) (lifetime risk: 1 in 6), with Black MSM (lifetime risk: 1 in 2) and Latino MSM (lifetime risk: 1 in 5) having greater risk than white MSM (lifetime risk: 1 in 11), illustrate compounded racial/ethnic and sexual minority disparities [6, 9]. Poverty further compounds this risk [4]. Among heterosexual individuals in high-poverty urban cities, the HIV prevalence rate is 20 times the overall prevalence among heterosexual people in the United States (US), indicating the powerful effect of place on HIV disparities [10]. Specifically, higher HIV rates are shown to be associated with county-level metrics of higher percentage of Black population and unemployment [7]. Improving HIV care continuum outcomes will require a focus on communities in high poverty areas which may in turn reduce HIV transmission [1, 11].

In Los Angeles County (LAC), Black and Latinx people accounted for 72% of new HIV diagnoses in 2018 (23% and 49%, respectively), and 65% of all people living with HIV (PLWH) as of 2019 (20% and 45%, respectively) [12]. Among all PLWH in LAC, 1 in 5 present with late-stage disease at diagnosis with CD4 <200 cells/mm^3^, but this rate is nearly 1 in 3 for Black and Latino cisgender men, and Latinx people in the US and LAC are more likely to receive late HIV testing compared to other racial and ethnic groups [12–14]. Black and Latinx PLWH also have lower rates of linkage to care, retention in care, and viral suppression when compared to white PLWH [12]. Overall there have been minimal to no improvements in the HIV care continuum in LAC from 2010–2019, with retention in care decreasing [12]. The high-poverty urban communities of LAC South Los Angeles (SLA) Service Planning Area 6 (SPA 6) have a majority of residents who are Black (27.4%) and Latinx (68.2%) [15]. These communities consistently have the second highest rates of new HIV diagnoses and PLWH of all LAC SPAs [12, 15, 16]. Furthermore, most SPA 6 communities are federally designated medically underserved areas, healthcare provider shortage areas, or both, with a third of residents reporting difficulty obtaining medical care when needed [17–19].

Further, LAC SPA 6 has geographical and historical significance, an area that includes Watts, Willowbrook and Compton. In the wake of the 1965 Watts Rebellion, the McCone Commission identified several leading community concerns related to socioeconomic disparities, such as inadequate health care services, housing and unemployment [20–23]. In response, the Martin Luther King Jr. Medical Campus, which has expanded to include Martin Luther King Jr. Outpatient Clinic (MLK OPC), was established in SPA 6 to address these health care needs. Given the significant HIV prevention and treatment disparities for Black and Latinx communities, and limited understanding of these outcomes in SPA 6, MLK OPC maintains the only public safety-net HIV care clinic in this setting, with ongoing research needed to guide the local HIV response [23, 24].

Therefore, to effectively address HIV prevention and treatment disparities in South Los Angeles, we conducted an exploratory study aimed at characterizing the care continuum among newly diagnosed individuals in this setting, and then outline implications for longitudinal studies to inform community-focused interventions.

## Methods

We conducted this exploratory study through secondary analysis of de-identified clinical medical record HIV data from MLK OPC, the only public safety-net clinic in SPA 6 located in the heart of the Watts, Willowbrook, Compton area of SLA. The outpatient center provides primary and specialty care, including HIV testing and treatment, for the under-resourced community. HIV testing in the adult primary care, women’s health, and urology departments is a routine opt-out per protocol order that nurses initiate on all new patients and any existing patients that do not have a test on record. The MLK OPC urgent care conducts routine opt-out testing for any individual getting a blood draw. MLK OPC routinely offers trainings on cultural competence, including LGBTQIA+ health care and gender-affirming care that is required of all staff.

### Data set

De-identified data including demographic, HIV testing, treatment outcomes were extracted from MLK OPC’s electronic medical record (EMR) system, Online Real-time Centralized Health Information Database (ORCHID). The study population includes all individuals who tested HIV positive at MLK OPC for the first time during 2016 through 2020, as these represent the first five years after test and treat/prevent guidelines were implemented. Specifically, the guidelines stressed two main points, antiretroviral therapy (ART) should be initiated in everyone diagnosed with HIV at any CD4 cell count and the use of daily oral pre-exposure prophylaxis (PrEP) is recommended as a prevention choice for people at substantial risk of HIV infection as part of combination prevention approaches [25].

Those who self-reported a prior diagnosis or with a baseline VL <200 copies/mL were excluded from the study. Initial data pull contained 436 individuals who tested positive and after a review of the baseline VL levels and clinical records, 155 participants were removed.

Demographic characteristics and zip codes were self-reported and recorded by clinical staff. Demographic variables include sex assigned at birth and self-identified gender identity, sexual orientation, age, race, ethnicity, primary language, employment status, and medical insurance type. The EMR system was used to extract the history of baseline viral load (VL), baseline CD4 count, and subsequent VL and CD4 counts.

The clinical data was cleaned by disaggregating responses, renaming variables for consistency, and coding categorical data for analysis. Baseline VL and CD4 counts were used to interpret disease progression at the time of diagnosis and prior to treatment. Los Angeles County Department of Public Health uses the following cut-offs to determine timeliness of HIV diagnosis and the stage of infection based on the first CD4 test result within 90 days of diagnosis: stage 1 is a CD4 ≥500 cells/μL, stage 2 is a CD4 between 200–499 cells/μL, and stage 3 is a CD4 <200 cells/μL. We used these cut-offs to capture timeliness of HIV diagnosis [12]. Dated VL and CD4 results were used to monitor testing and treatment outcomes. Time to linkage to care was measured by calculating the time between the positive test and first VL in the EMR, as the first VL test was used as a proxy for confirming linkage to care. Time between positive test and virally suppressed and/or undetectable VL was calculated to measure treatment outcomes of viral suppression, undetectability, and viral rebound. The sensitivity of the LAC Division of HIV and STD Programs VL monitor tests is 40 copies/mL, which we defined as the undetectable limit. Viral suppression was defined as <200 copies/mL.

### Data analysis

Descriptive and bivariate analyses were performed. Pearson Chi-square and Fisher’s Exact test were used to explore the association between demographic characteristics and HIV outcomes. A Kaplan-Meier survival curve was used to present the time to linkage to care. This analysis shows the percentage of patients that have yet to be linked at each time point following a positive diagnosis [26]. Because this was an exploratory analyses with a small sample size, and we did not have specific outcome variables of interest, multivariate analyses were not performed. All reported p-values were two sided; and p<0.05 was considered statistically significant. SPSS for windows version 26.0 was used for the analysis (IBM SPSS Inc., New York, USA).

### Ethics

This study received ethical approvals from the Los Angeles County Department of Public Health Ambulatory Care Network and Health Services Institutional Review Board and Charles R. Drew University Institutional Review Board. As this was a retrospective study of medical records, a waiver of informed consent was granted as the research had no more than minimal risk to participants and could not be carried out without the waiver. All data was de-identified and fully anonymized prior to the research team having access.

## Results

From 2016 to 2020, 281 individuals were identified as newly diagnosed at MLK Outpatient Center. Per year, 63 (22.4%) were diagnosed in 2016, 55 (19.6%) in 2017, 64 (22.8%) in 2018, 49 (17.4%) in 2019, and 50 (N = 17.8) in 2020. Descriptive analysis of socio-demographic characteristics is shown in Table 1. We identified males (n = 239, 85.1%) and females (n = 42, 14.9%), based on assigned sex at birth. Of those reporting their gender identity (n = 186), the majority identified as cisgender men (n = 151, 81.2%), followed by cisgender women (n = 49, 15.6%), transgender women (n = 4, 2.2%), and transgender men (n = 2, 1.1%). There was a mean age of 36 years, median of 34, and a range of 13–72 years. The most common self-identified race/ethnicity was Black (132, 48.2%), followed by Hispanic/Latinx (n = 109, 39.8%), other race (n = 29, 10.6%), and white (n = 4, 1.5%). Self-reported sexual orientation shows straight (n = 89, 40.5%), gay (n = 95, 43.2%), bisexual (n = 32, 14.5%), and lesbian (n = 4, 1.8%) individuals among the newly diagnosed. The majority of clients were unemployed (N = 145, 66.2%) and primary languages were English (n = 208, 75.4%), Spanish (n = 66, 23.9%), and other (n = 2, 0.7%).

**Table 1:** Demographic characteristics of newly diagnosed HIV-positive clients in 2016–2020 (N = 281).

	N	Valid %
**Assigned sex at birth (N = 281)**		
Male	239	85.1
Female	42	14.9
**Self-identified gender identity (N = 186)**		
Cisgender Man	151	81.2
Cisgender Woman	29	15.6
Transgender Woman	4	2.2
Transgender Man	2	1.1
**Age group (N = 280)**		
<20	12	4.3
20–29	92	32.9
30–39	80	28.6
40–49	52	18.6
50–59	30	10.7
≥ 60	14	5
**Race/Ethnicity (N = 274)**		
Black/African American	132	48.2
Latinx/Hispanic	109	39.8
White	4	1.5
Other	29	10.6
**Self-identified sexual orientation (N = 220)**		
Straight	89	40.5
Gay	95	43.2
Lesbian	4	1.8
Bisexual	32	14.5
**Employment status (N = 219)**		
Unemployed	145	66.2
Employed	70	32
Disabled	4	1.8
**Primary language (N = 276)**	208	75.4
English	66	23.9
Spanish	2	0.7
Other		
**Insurance Coverage (N = 278)**		
Medi-Cal	169	60.8
Ryan White Coverage	31	11.2
Self-Pay Outpatient	51	18.3
MediCare	15	5.4
Other	12	4.3

Baseline CD4 outcomes by socio-demographic characteristics are shown in Table 2. Of those who had a baseline CD4 (n = 255), the distribution was ≤ 200 (n = 100, 39.2%), 201–499 (n = 89, 34.9%), and ≥ 500 (n = 66, 25.9%). Age (p = 0.002), race/ethnicity (p = 0.008), employment status (p = 0.031), and primary language (p = 0.026) were significantly related to baseline CD4 count.

**Table 2:** Baseline CD4 of newly diagnosed HIV-positive clients in 2016–2020 by demographic characteristics, excluding white (N = 281).

Baseline CD4 Count	≤ 200	201–499	≥ 500	
	N(%)	N(%)	N(%)	P value
**Assigned sex at birth (N = 281)**				
Male	66 (37.1)	64 (36.0)	48 (27.0)	0.999
Female	10 (35.7)	11 (39.3)	7 (25.0)	
**Self-identified gender identity (N = 186)**				
Cisgender Man	42 (39.3)	37 (34.6)	28 (26.2)	0.848
Cisgender Woman	4 (25.0)	8 (50.0)	4 (25.0)	
Transgender Woman	1 (25.0)	1 (25.0)	2 (50.0)	
Transgender Man	1 (50.0)	1 (50.0)	0 (0.0)	
**Age group (N = 280)**				
<20	0 (0.0)	4 (50.0)	4 (50.0)	0.002
20–29	13 (18.6)	32 (45.7)	25 (35.7)	
30–39	29 (47.5)	16 (26.2)	16 (26.2)	
40–49	20 (54.1)	11 (29.7)	6 (16.2)	
50–59	10 (50.0)	8 (40.0)	2 (10.0)	
≥ 60	4 (40.0)	4 (40.0)	2 (20.0)	
**Race/Ethnicity (N = 274)**				
Black/African American	37 (34.3)	41 (38.0)	30 (27.8)	0.009
Latinx/Hispanic	38 (48.7)	23 (29.5)	17 (21.8)	
Other	1 (5.0)	11 (55.0)	8 (40.0)	
**Self-identified sexual orientation (N = 220)**				
Straight	19 (32.8)	21 (36.2)	18 (31.0)	0.240
Gay	29 (38.2)	24 (31.6)	23 (30.3)	
Lesbian	0 (0.0)	11 (45.8)	5 (20.8)	
Bisexual	8 (33.3)	3 (100.0)	0 (0.0)	
**Employment status (N = 219)**				
Unemployed	47 (42.0)	38 (33.9)	27 (24.1)	0.031
Employed	12 (25.5)	17 (36.2)	18 (38.3)	
Disabled	3 (100.0)	0 (0.0)	0 (0.0)	
**Primary language (N = 276)**				
English	64 (34.0)	69 (36.7)	55 (29.3)	0.026
Spanish	33 (55.9)	19 (32.2)	7 (11.9)	
Other	1 (50.0)	0 (0.0)	1 (50.0)	
**Insurance Coverage (N = 278)**				
Medi-Cal	41 (36.5)	37 (32.3)	36 (31.3)	0.799
Ryan White Coverage	9 (31.0)	14 (48.3)	6 (20.7)	
Self-Pay Outpatient	19 (43.2)	166 (36.4)	9 (20.5)	
MediCare	3 (3.33)	4 (44.4)	2 (22.2)	
Other	2 (28.6)	3 (42.9)	2 (28.6)	

**Fig 1:**
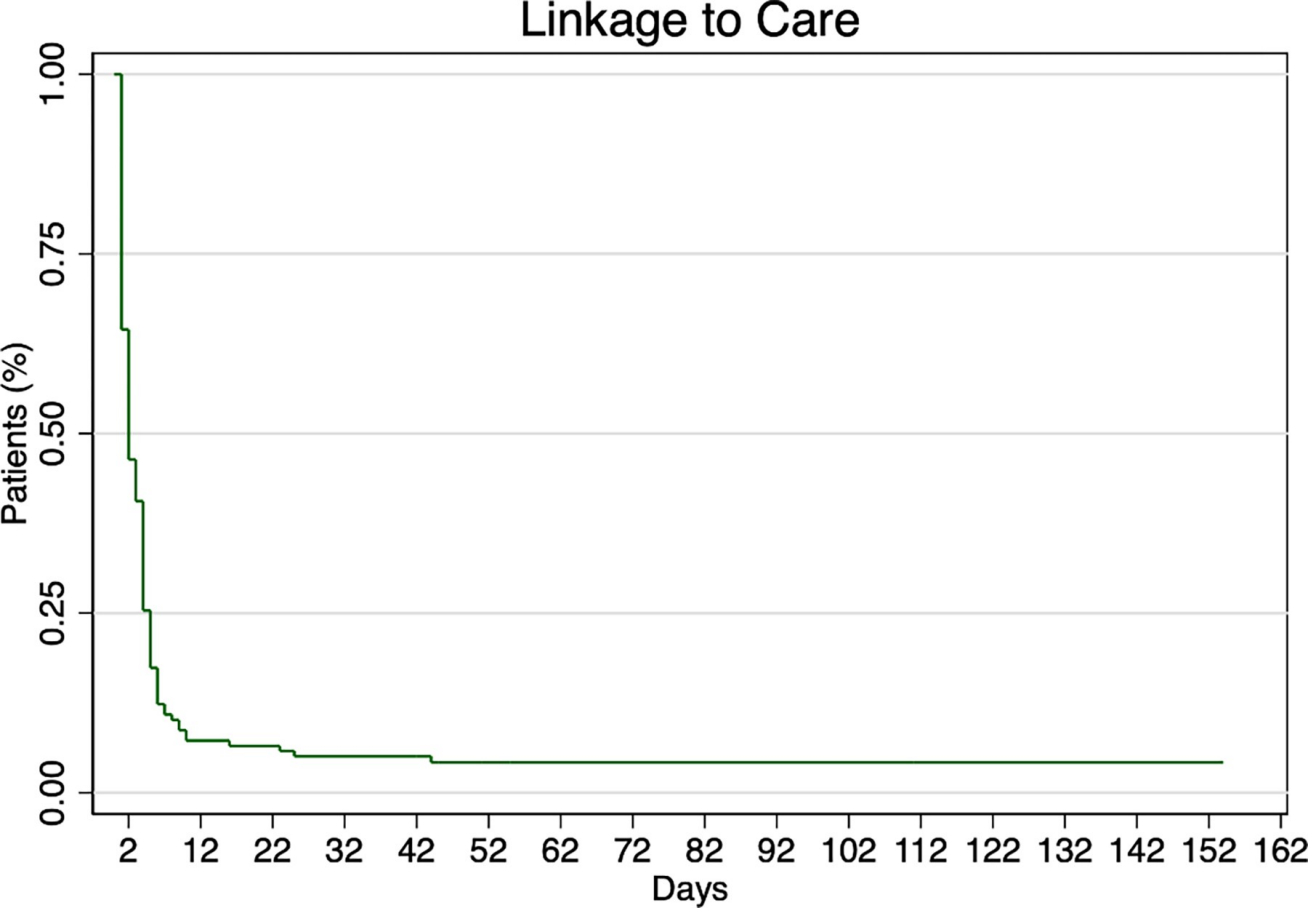
Linkage to care. Kaplan-Meier curve for the outcome linkage to care, as measured by the date of the first documented VL result in the EMR. Time in days is calculated from the date of the first positive HIV test. The curve shows the percent of patients that do not yet have a VL result at each time point.

The Kaplan-Meier curve (Fig 1) shows the trajectory of patient linkage to care (measured by the date of a documented VL result in the EMR). Since all patients were newly diagnosed, at the time of the positive test, all patients had yet to be linked. The median time to linkage, as indicated by the time at which 50% had linked, was approximately 3 days. By 30 days, only 10% had yet to be linked. Other HIV care continuum outcomes by race/ethnicity are shown in Table 3. Among all newly diagnosed individuals, 90% were linked to care, and 85.1% were prescribed antiretroviral therapy. Individuals diagnosed in 2020 were excluded from the rest of care continuum outcomes. Achievement of viral suppression, VL<200 (n = 131, 59.8%), and undetectable viral load, VL <40 (n = 113, 52.6%), were low overall and lower among Black clients (n = 61, 54.5% and n = 54, 49.1% respectively). The median times to viral suppression and undetectability were 77 days and 121 days, respectively. Only 42.4% of clients were retained in care, defined as 2 or more VLs ≥ 3 months apart in a year, but retention was significantly higher among Latinx clients (51.2%, p = 0.036). Of those who achieved viral suppression, 20.7% (n = 25) experienced viral rebound, with a subsequent VL >200 within the calendar year following diagnosis. We classified individuals as lost to follow up (n = 20, 9.3%) if they had zero or one VL on record.

**Table 3:** HIV care continuum outcomes of newly diagnosed HIV-positive clients in 2016–2020 by race/ethnicity, excluding white (N = 281).

	Black	Latinx	Other Race	Total*	
	N (Valid %)	N (Valid%)	N (Valid%)	N (Valid%)	P value
**Linked to Care**	105 (93.8)	76 (92.7)	17 (68.0)	198 (90.4)	0.000
**ART Prescription**	107 (81.7)	97 (89.8)	23 (79.3)	227 (84.7)	0.153
**Viral Suppression**	61 (54.5)	58 (70.7)	12 (48.0)	131 (59.8)	0.111
**Undetectable**	54 (49.1)	47 (58.8)	12 (48.0)	113 (52.6)	0.226
Retained in Care**	47 (42.0)	42 (51.2)	8 (32.0)	97 (44.3)	0.185
**Viral Rebound**	13 (22.8)	9 (17.3)	3 (15.0)	25 (20.7)	0.721
**Lost to Follow-Up**	10 (9.1)	5 (6.3)	5 (20.0)	20 (9.3)	0.226

* Total is excluding white.

## Discussion

Our exploratory study identified newly diagnosed HIV positive individuals from highly impacted minority groups and revealed differences along the care continuum between LAC and SPA 6, an underserved and understudied area. We found two times as many Black individuals in our study population compared to LAC, reflecting both demographic differences between SPA 6 and LAC, with 27.4% of Black residents and 12.6% of Black residents respectively, and the disproportionate burden of HIV on the Black community [15]. While Black residents account for 27.4% of the SPA 6 population, they accounted for 48.2% of the newly diagnosed in our study population.

Not all clients had data on gender identity and sexual orientation in the EMR, which limited our ability to fully identify and analyze sexual and gender minority representation. It is unclear if this missing data is due to a lack of self-reporting or failure of staff to collect; however, research shows that LGBTQIA+ individuals often withhold personal health information related to sexual orientation due to previous negative interactions, perception of healthcare settings and providers as threatening, and concerns over the quality of healthcare they will receive after disclosing [27]. Although there are LGBTQIA+ competence trainings implemented at the MLK OPC, sustained efforts on the part of healthcare providers to build relationships with LGBTQIA+ clients will be important to reaffirm welcoming clinic environments which will in turn improve disclosure and HIV care [28].

The majority of those whose data was available identified as cisgender men (81.2%) and 43.2% identified as gay. Our overall 40.5% representation of heterosexual people is supported by national data that shows a 20 times greater HIV prevalence among heterosexual people in urban areas with high-poverty in the US [10]. Further illustrating an association between HIV prevalence and socioeconomic disadvantage, two-thirds of the newly diagnosed clients reported unemployment and 60.8% were covered by Medi-Cal insurance.

The percentage of clients with baseline CD4 count <200 (39.2%) is more than twice the national goal (16.0%) and higher than the LAC rate (22.0%). Altogether, 74.1% of clients presented with baseline CD4 count <500 [12]. These high percentages of low CD4 counts demonstrate late diagnoses and indicate a need for increased routine testing for early diagnosis. The increased likelihood of a CD4 <200 for those unemployed is also supported by findings of neighborhood level unemployment being associated with late diagnosis and delayed presentation [29, 30]. While nearly all newly diagnosed individuals were linked to care within 30 days—measured by time from positive HIV test to first VL in the EMR—surpassing LAC and national linkage to care goals, this successful linkage to care is not reflected in high rates of viral suppression, undetectability, and retention in care, despite the introduction of a newer single tablet regimen in 2015 [31].

Importantly, of those who did reach viral suppression, 1 in 5 experienced viral rebound within the calendar year following diagnosis. This is 2.76 times national estimates of viral rebound prevalence [32]. Disparities in viral rebound exist alongside other care continuum disparities, with higher prevalence found among several subpopulations including Black people, those recently experiencing homelessness, and people with public insurance [32]. Currently, LAC Annual Surveillance Reports do not include information on viral rebound, but our results show that this may be a necessary outcome to measure in working towards ending HIV transmission and improving HIV treatment outcomes, especially among racial, ethnic, sexual, gender, and class minorities.

As this study utilized data from the EMR to analyze testing and treatment outcomes, further research will be needed to understand outcomes for those lost-to-care along the continuum. Additionally, analysis was only conducted on data from the MLK OPC EMR system, and did not incorporate data from EMRs of other clinics; thus failing to capture outcomes for individuals who may have continued their treatment elsewhere.

### Implications

Early HIV diagnosis, retention in care, and sustained viral suppression remain considerable public health challenges which must be addressed for optimal individual treatment outcomes, successful treatment-as-prevention, and reduced HIV disparities [1, 33]. This demonstrates the need to understand community viral loads to determine burden of viremia to compare across SPAs, specifically among Black and Latinx subpopulations. Thus, longitudinal studies are needed to characterize HIV care continua because they provide more accurate insight into long-term outcomes than do cross-sectional studies, which may overestimate successful treatment outcomes, and expose disparities in care continua [33]. Such analyses should be run at the community level, especially in high poverty urban areas and areas with high concentrations of Black and Latinx residents, to understand the unique barriers faced by these communities and to develop interventions that effectively respond to local HIV epidemics.

## Conclusion

Characterizing HIV care continua by race, ethnicity, sexual and gender identity, and socioeconomic standing emphasizes the importance of place-based responses to address disparities. Utilizing community collaborations, clinics can gather and analyze local data regularly to help inform evidence-based, data-informed public health responses to end HIV transmission and improve health and wellness for those living with HIV in high poverty areas.
